# Protective Effect of Uridine on Structural and Functional Rearrangements in Heart Mitochondria after a High-Dose Isoprenaline Exposure Modelling Stress-Induced Cardiomyopathy in Rats

**DOI:** 10.3390/ijms242417300

**Published:** 2023-12-09

**Authors:** Natalia V. Belosludtseva, Lubov L. Pavlik, Irina B. Mikheeva, Eugeny Yu. Talanov, Dmitriy A. Serov, Dmitriy A. Khurtin, Konstantin N. Belosludtsev, Galina D. Mironova

**Affiliations:** 1 Institute of Theoretical and Experimental Biophysics, Russian Academy of Sciences, Institutskaya 3, 142290 Pushchino, Russia; pavlikl@mail.ru (L.L.P.); mikheirina@yandex.ru (I.B.M.); evg-talanov@yandex.ru (E.Y.T.); bekonik@gmail.com (K.N.B.); 2 Prokhorov General Physics Institute, Russian Academy of Sciences, Vavilov St. 38, 119991 Moscow, Russia; dmitriy_serov_91@mail.ru; 3 Department of Biochemistry, Cell Biology and Microbiology, Mari State University, pl. Lenina 1, 424001 Yoshkar-Ola, Russia; dimaxur11@mail.ru

**Keywords:** isoprenaline exposure, mitochondrial dysfunction, mitochondrial morphology, uridine, ATP synthesis, ROS, cardioprotection

## Abstract

The pyrimidine nucleoside uridine and its phosphorylated derivates have been shown to be involved in the systemic regulation of energy and redox balance and promote the regeneration of many tissues, including the myocardium, although the underlying mechanisms are not fully understood. Moreover, rearrangements in mitochondrial structure and function within cardiomyocytes are the predominant signs of myocardial injury. Accordingly, this study aimed to investigate whether uridine could alleviate acute myocardial injury induced by isoprenaline (ISO) exposure, a rat model of stress-induced cardiomyopathy, and to elucidate the mechanisms of its action related to mitochondrial dysfunction. For this purpose, a biochemical analysis of the relevant serum biomarkers and ECG monitoring were performed in combination with transmission electron microscopy and a comprehensive study of cardiac mitochondrial functions. The administration of ISO (150 mg/kg, twice with an interval of 24 h, s.c.) to rats caused myocardial degenerative changes, a sharp increase in the serum cardiospecific markers troponin I and the AST/ALT ratio, and a decline in the ATP level in the left ventricular myocardium. In parallel, alterations in the organization of sarcomeres with focal disorganization of myofibrils, and ultrastructural and morphological defects in mitochondria, including disturbances in the orientation and packing density of crista membranes, were detected. These malfunctions were improved by pretreatment with uridine (30 mg/kg, twice with an interval of 24 h, i.p.). Uridine also led to the normalization of the QT interval. Moreover, uridine effectively inhibited ISO-induced ROS overproduction and lipid peroxidation in rat heart mitochondria. The administration of uridine partially recovered the protein level of the respiratory chain complex V, along with the rates of ATP synthesis and mitochondrial potassium transport, suggesting the activation of the potassium cycle through the mitoK_ATP_ channel. Taken together, these results indicate that uridine ameliorates acute ISO-induced myocardial injury and mitochondrial malfunction, which may be due to the activation of mitochondrial potassium recycling and a mild uncoupling leading to decreased ROS generation and oxidative damage.

## 1. Introduction

According to the World Heart Federation 2023 report, cardiovascular diseases are responsible for one third of all deaths globally and remain a major problem in medicine worldwide despite advances in their management in recent years [1]. Among cardiovascular diseases, ischemic cardiomyopathies are the most widespread and severe myocardial pathologies, which are characterized by resistance to ongoing therapy and high mortality [2]. A separate group of cardiomyopathies includes stress-induced cardiomyopathies associated with severe left ventricular dysfunction, which is caused by a profound catecholamine surge without the involvement of coronary artery occlusion [3,4]. The acute phase of the condition is the most dangerous and can lead to lethal complications, including fatal ventricular arrhythmias and cardiogenic shock. Adrenergic overstimulation plays an essential role in the initiation of this pathology and can cause functional hypoxia and ischemia, leading to cardiac cell damage, but the pathophysiological pathways involved are not well understood. Moreover, there are no recommendations for the management of the pathology, and the use of the same therapeutic approaches as for acute coronary events may worsen the condition of such patients [5,6,7].

One of the most reliable and simple models for mimicking stress-induced cardiomyopathies is a rat model of acute subcutaneous overdosing of the synthetic β-adrenergic agonist isoprenaline (ISO) [8,9,10,11]. A hyperactivation of β-adrenoreceptors of rat cardiomyocytes by ISO in high doses leads to left ventricular dysfunction with ballooning and ischemia–reperfusion injury, which is known to involve oxidative stress, proinflammatory responses, calcium overload, mitochondrial malfunction, and, ultimately, cell death [10,11,12]. Nevertheless, the subcellular mechanisms involved in ISO-induced acute ischemic damage beyond adrenergic overstimulation are still poorly understood [13,14]. Although alterations in mitochondrial function have been identified [10,12,15,16], they have not been thoroughly studied, especially in the acute period.

Research in recent decades has revealed that mitochondria act as ‘sensors’ of cellular stress and play a central role in heart health and disease. Heart muscle fibers contain a large number of mitochondria, known for their role in ATP synthesis through oxidative phosphorylation. A sharp decline in mitochondrial oxidative respiration and ATP generation leads to a state of energy deficit in the myocardium and disrupts the energy-consuming process of cardiac muscle contraction [17]. Along with dramatic changes in energy metabolism, mitochondrial malfunction can lead to a dysregulation of cellular redox homeostasis. It is known that, under ischemia injury and toxin exposure, mitochondria may become a significant generator of pathological amounts of ROS through the electron transport chain and other sites [18,19,20,21]. In addition to these functions, mitochondria can serve a critical function in the maintenance of ionic (mainly calcium and potassium ion) homeostasis, be involved in inflammatory responses, release pro-apoptotic signals via the opening of the permeability transition pore (mPTP), leading to the loss of the mitochondrial membrane potential and eventual cell death, modulate nuclear gene expression, and synthesize essential macromolecules, such as lipids, proteins, DNA, and RNA [22,23]. The ultrastructure of mitochondria, particularly their highly conserved inner membrane cristae, is mechanistically linked to all these functions, and vice versa [24]. Since cardiac mitochondria comprise 25–30% of the total cell volume, their ultrastructural and functional abnormalities in the myocardium mediated by various molecular mechanisms make a significant contribution to the development of cardiovascular pathologies and enhanced oxidative stress and, therefore, are an attractive pharmacological target for therapeutic intervention [19,20,21].

Uridine, a pyrimidine nucleoside containing uracil and ribose, is mostly known as a precursor for RNA; however, this nucleoside and its derivatives also play an essential role in the regulation of energy balance and oxidative phosphorylation [25,26], systemic metabolism [25], glycogen synthesis [27,28], membrane integrity, and normal cardiac function [29,30,31]. Recent insights into global transcriptomic and proteomic profiling in murine models suggest that uridine can promote the process of tissue repair and regeneration by modulating the metabolic processes [32]. It is known that the concentration of circulating plasma uridine is tightly controlled to ensure proper cell functions [25], and it can decrease dramatically upon myocardial ischemia [33]. Our studies have shown that uridine exerts protective effects in rat models of hypoxic hypoxia [34], lipopolysaccharide-stimulated inflammation [33], diabetic cardiomyopathy [35], and local circulatory hypoxia (acute myocardial ischemia by the occlusion of the left coronary artery) by activating the mitochondrial ATP-dependent potassium (mitoK_ATP_) channel [30]. This assures the preservation of the morphology and function of mitochondria and, thus, recovers the energy supply and reduces oxidative stress in cells. Some studies indicate that uridine supplementation can modulate the activity of the electron transport chain through the mitochondrial membrane-bound and respiratory complex-coupled enzyme dihydroorotate dehydrogenase [36,37] and preserve the levels of Krebs cycle intermediates and other mitochondrial metabolites in a cellular model of oxidative phosphorylation dysfunction [26].

Taking the aforesaid into account, the aim of this work was to evaluate the cardioprotective potential of uridine (30 mg/kg body wt., i.p.) against acute cardiac injury at an early (48 h) time point after exposure to ISO (150 mg/kg body wt., s.c.) and to investigate the effect of the nucleoside on the development of structural and functional disorders of heart mitochondria in a rat model of stress-induced cardiomyopathy.

## 2. Results

### 2.1. Study Design and Baseline Characteristics of Experimental Animals

**Figure 1:**
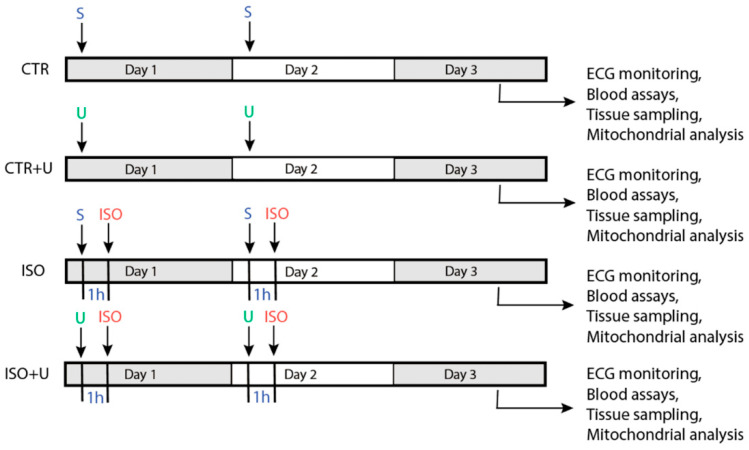
Study design and analyses. Four groups of experimental rats were included in the study: control (CTR, *n* = 10), control + uridine (CTR + U, *n* = 10), isoprenaline hydrochloride (ISO, *n* = 13), isoprenaline + uridine (ISO + U, *n* = 11). S, saline solution.

**Figure 2:**
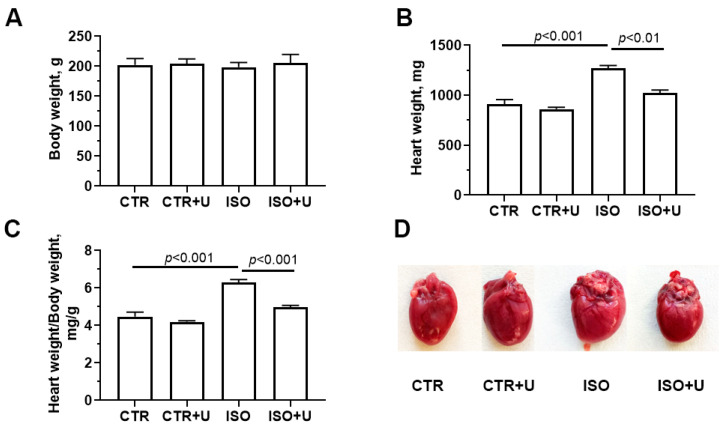
Baseline characteristics of experimental animals. Body weight (**A**), heart weights in absolute values (**B**) and relative to body weight (**C**), and typical photographs of isolated hearts (**D**) of Wistar rats in four experimental groups: control (CTR), control + uridine (CTR + U), isoprenaline hydrochloride (ISO), isoprenaline + uridine (ISO + U). The representative image of an isolated heart in the ISO group shows the characteristic sign of ballooning of the apex of the left ventricle. The values are the means, with standard errors represented by vertical bars (*n* = 10). One-way ANOVA followed by Tukey’s post hoc test was used.

Figure 1 and Figure 2 show the study design and the analyses of the acute effects of ISO and uridine on the baseline somatic indicators of animals at the end of the experiment. For this study, male Wistar rats (*n* = 44) were randomly divided into four groups: the control (CTR) group received two intraperitoneal injections of physiological saline (S) with an interval of 24 h; the control + uridine (CTR + U) group was injected with uridine according to the same scheme (U, 30 mg/kg body wt., i.p.); the isoprenaline (ISO) group was treated with two repeated injections of isoprenaline hydrochloride at a dose of 150 mg/kg body wt., s.c., with a 24h interval; the isoprenaline + uridine (ISO + U) group was injected with uridine (30 mg/kg body wt., i.p.) 1 h before each injection of ISO. Rats were sacrificed 48 h after the first injection, and the assays listed were performed.

Heart weight in absolute values was defined by weighing the blood-free organ from an experimental rat, and the relative heart weight was determined using the formula: heart weight (HW) (mg)/body weight (BW) (g) (Figure 2). Experimental animals showed no difference in body weight. However, ISO administration increased both the absolute heart weight and heart weight normalized to body weight by 28% and 40% compared to the control, respectively, due to an increase in mechanical stress and in response to an increase in neurohormonal stimulation [38,39]. Pretreatment with uridine significantly reduced the increase in heart weight and the HW/BW coefficient (*p* < 0.05).

Regarding the mortality, there were no deaths in the control groups (CTR and CTR + U), while the mortalities in the ISO and ISO + U groups were 30% and 10%, respectively.

### 2.2. Electrophysiological Changes in the Hearts of Experimental Rats

**Figure 3:**
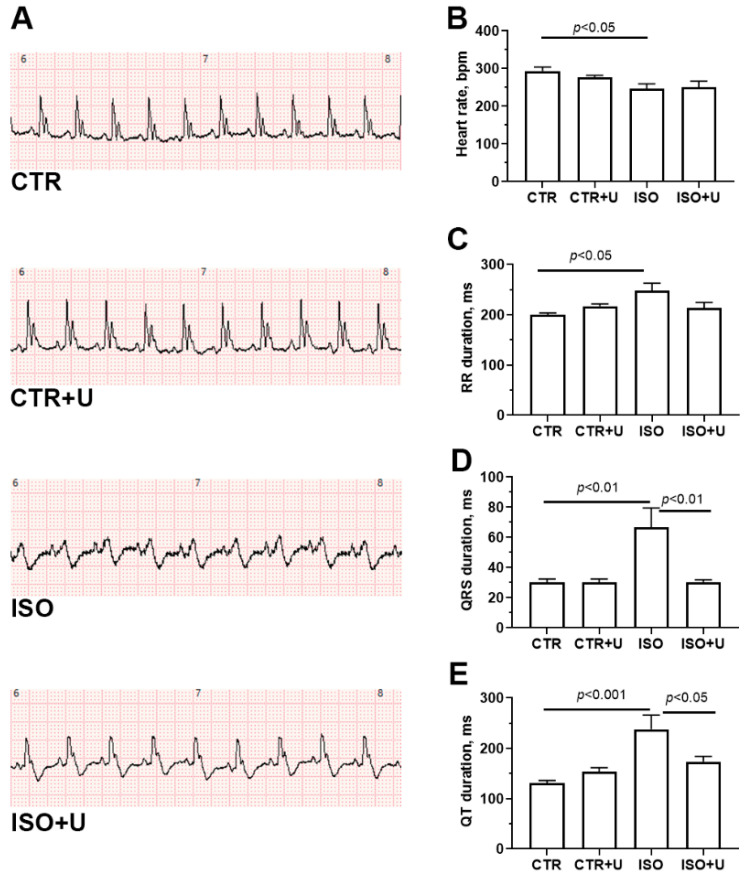
Lead II electrocardiogram (ECG) trace patterns (**A**), heart rate (beats per minute) (**B**), and the most common ECG intervals: RR (**C**), QRS complex (**D**), and QT (**E**) intervals (in milliseconds) in the studied animal groups (C, control; C + U, control + uridine; ISO, ISO-induced cardiomyopathy; U + ISO, uridine + isoprenaline-induced cardiomyopathy). Data are expressed as mean values ± SEM from a group of five animals. Statistical significance was analyzed using one-way ANOVA followed by Tukey’s post hoc test.

To verify the cardiac responses of experimental rats to drug interventions, electrocardiographic (ECG) monitoring was carried out. Figure 3A shows typical lead II ECG trace patterns in each experimental group of rats. An analysis of ECG recordings (Figure 3B,C) revealed that the administration of high-dose ISO to animals caused a decrease in heart rate and a lengthening of the RR interval, which is typical for this type of lesion and reflects an overall impairment of myocardial contractility and relaxation [40,41,42,43]. Moreover, there was a statistically significant increase in the duration of the QRS complex and the QT interval (*p* < 0.05). In addition, an inversion of the T wave and a decrease in the amplitude of the R wave were observed in the ISO group. Pretreatment with uridine at a dose of 30 mg/kg for two consecutive days, 1 h before each injection of ISO, led to partial normalization of the QT interval, but it did not significantly affect other ECG intervals in ISO-injected rats (Figure 3B). In control rats that received uridine alone, no changes were observed in the most common ECG intervals.

### 2.3. Effect of Uridine Pretreatment on Serum Cardiac Biomarkers and ATP Content in Heart Tissue Homogenates of Rats after Acute Exposure to ISO

**Figure 4:**
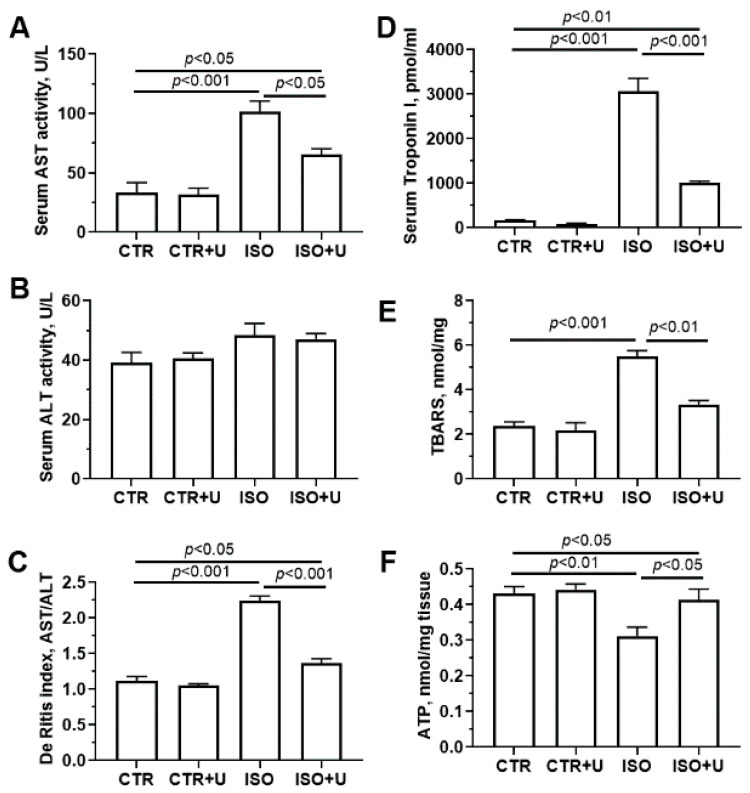
Blood serum activity of aspartate transferase (AST) (**A**), alanine transaminase (ALT) (**B**), AST/ALT (De Ritis) ratio (**C**), serum cardiac specific troponin I concentration (**D**), serum levels of thiobarbituric acid reactive substances (TBARS) (**E**), and ATP content in tissue homogenates of the left ventricle (**F**) from experimental rats. Data are expressed as mean values ± SEM from a group of five animals. Statistical significance was analyzed using one-way ANOVA followed by Tukey’s post hoc test.

Then, we investigated the changes in biochemical markers of myocardial damage in the blood serum of experimental rats, including the activity of the enzymes aspartate transaminase (AST) and alanine transaminase (ALT), and the concentrations of cardiac troponin I and thiobarbituric acid (TBA) reactive substances (TBARS) (Figure 4). It was found that after administration of ISO, cardiac troponin I concentration in the serum of animals increased more than 30-fold compared to that in vehicle-treated rats. When uridine was administered to ISO-treated animals, the level of serum troponin I decreased by 67%. The activity of serum AST, a classical marker of cardiac injury, was increased threefold in the ISO group, whereas the activity of serum ALT in this group only tended to increase and was not statistically different from baseline values in the control group. These changes were accompanied by a significant (2.5-fold) increase in the AST/ALT ratio (De Ritis ratio) in ISO-treated animals. Furthermore, ISO administration markedly (by 40%) increased the content of serum TBA-reactive substances, the byproducts of lipid peroxidation. In the ISO + U group, serum AST activity, the De Ritis coefficient, and the level of serum TBA-reactive substances were lower compared with the ISO group (*p* < 0.05). It should be noted that in the CTR + U group, no difference in these indices was observed compared to the CTR group.

To assess changes in the energy status of the myocardium of experimental rats, the ATP content in left ventricle tissue was measured (Figure 4F). It was found that the administration of ISO to rats led to a decrease in the level of ATP in tissue samples by 30% compared to the control, which suggests a dysregulation of energy metabolism in the myocardium in the experimental setting. When uridine was injected into ISO-treated rats, the myocardial ATP content increased by 16%.

### 2.4. Effect of Uridine Administration on Ultrastructural Alterations in Mitochondria of Left Ventricular Cardiomyocytes from ISO-Treated Rats

**Figure 5:**
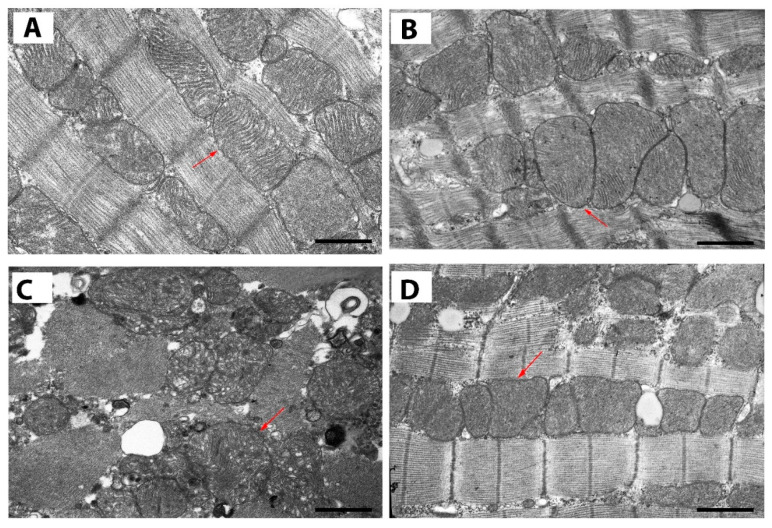
Transmission electron microscopy (TEM) images (2500×) showing representative examples of mitochondria in cardiomyocytes of the left ventricles of rats from four experimental groups: (**A**) Control; (**B**) Control + Uridine; (**C**) ISO; (**D**) ISO + Uridine. Arrows indicate typical mitochondria located between the myofibrils. Uranyl acetate and lead citrate × 2500. Scale bars, 1 μM.

In order to determine the structural changes in rat cardiomyocytes from experimental groups, tissue samples from identical regions of the left ventricle (apical segments) were taken and examined using transmission electron microscopy (TEM) analysis. Figure 5 shows representative electron micrographs of sections of cardiomyocytes of the left ventricles of the hearts of rats from four experimental groups. One can observe a normal architecture of heart mitochondria along with a good appearance of myofilaments in the control group. In the group treated with uridine (CTR + U), no pathological changes in mitochondrial structure and myofilaments are visible. In these two groups, mitochondria are arranged in rows between myofibrils, closely adjacent to each other, and have a similar round shape. By contrast, the ISO group shows a disturbance of the normal parallel arrangement of the mitochondria, vacuolization, occurrence of pathologically shaped organelles, along with impairment of packing density, arrangement order, and the orientation of the crista membranes. In addition, there are abnormalities in the organization of sarcomeres with focal disorganization of myofibril packing compared with the control. In ISO + U animals, mitochondrial shape and cristae architecture are largely preserved. Myofibrillar disorganization and vacuolization in this group are less pronounced.

**Figure 6:**
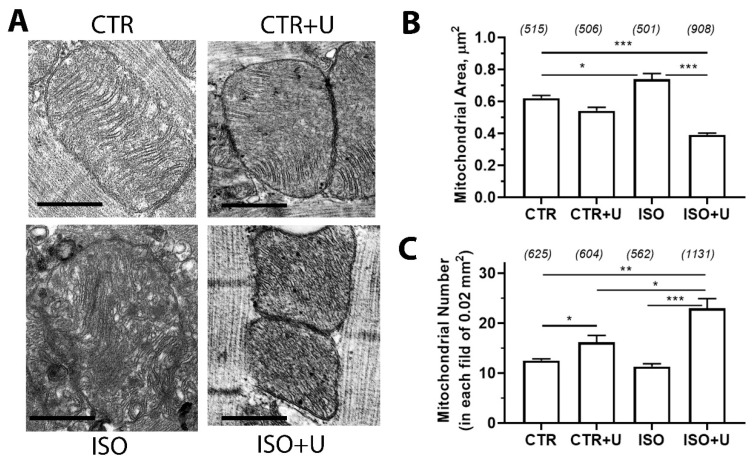
Ultrastructural alterations in rat heart interfibrillar mitochondria from four experimental groups. (**A**) Microphotographs of individual interfibrillar mitochondria in cardiomyocytes from experimental rats. Scale bars, 1 μM. (**B**) Bar graph summarizing the average data of the mitochondrial area (μm^2^) between conditions (the number of mitochondria counted are shown in parentheses). (**C**) Average number of mitochondria in rat cardiomyocytes, normalized to the area (mm^2^) of the pictures analyzed. Data are given as means ± SD. At least 30 fields of view were processed in each group. The analyses were performed from at least three rat left ventricles per group. * *p* < 0.05; ** *p* < 0.01; *** *p* < 0.001.

A morphometric analysis of the parameters of individual mitochondria from four experimental groups is shown in Figure 6. Compared to the control, the area of mitochondria in the ISO group was higher, while the number of mitochondria did not change significantly. In the ISO + U group, a significant increase in the mitochondrial number compared to the ISO animals was found, which may be due to a decrease in the area of mitochondria. Noteworthy, uridine supplementation to control animals also led to an increase in the number of mitochondria per unit area.

### 2.5. Effect of Uridine Administration on Mitochondrial Respiration, Oxidative Phosphorylation, and mtDNA Level in Experimental Rats

Table 1 shows that the exposure to ISO was accompanied by a decrease in the rate of oxygen consumption by rat heart mitochondria in the ADP-stimulated (phosphorylating) state 3 (V_3_) and DNP-stimulated (uncoupled) state 3U_DNP_ (V_DNP_) by 32 and 30%, respectively, compared with the control, in the presence of substrates of the respiratory chain complex I malate (2.5 mM) and glutamate (2.5 mM). Similar changes, but less pronounced, were found when succinate (5 mM) was used as a substrate of mitochondrial respiratory complex II (Table S1). These changes were accompanied by a significant decrease in the respiratory control ratio (RCR) and the ADP/O coefficient, as well as an increase in the time of ADP phosphorylation (T_ph._) in the ISO group compared with the control.

In the ISO + U group, the respiratory rates of mitochondria in states 3 and 3U_DNP_ were increased compared with the ISO group (Table 1). As a result, the RCR and the ADP/O coefficient were higher, and the phosphorylation time was reduced compared with these parameters for the group of ISO-treated animals. It should be mentioned that uridine alone had no effect on the efficiency of oxidative phosphorylation (OXYPHOS) when using NAD- and FAD-dependent respiratory substrates in control animals.

**Figure 7:**
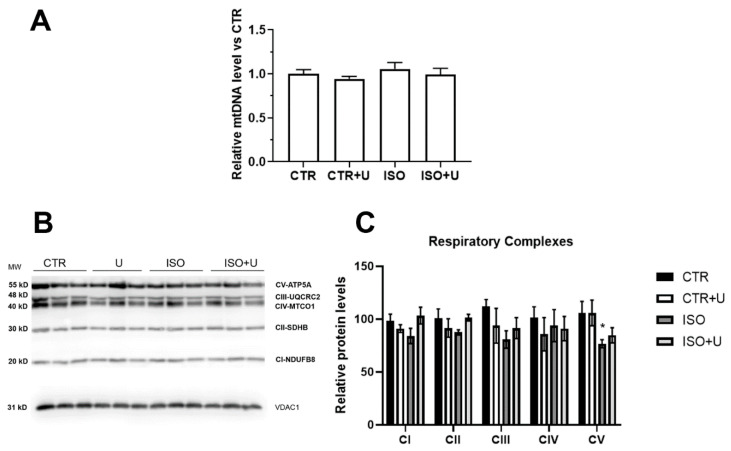
Relative levels of mtDNA and a subset of oxidative phosphorylation (OXPHOS) subunits in rat heart mitochondria from four experimental groups. (**A**). Relative amount of the mtDNA copy number in the heart tissue of four animal groups. The relative copy number of each group was calculated as the ratio between the mtDNA and nuclear DNA copy numbers. Data are expressed as mean values ± standard error from six independent experiments. (**B**). Representative Western blot of typical subunits of complexes I–V of the respiratory chain (CI-NDUFB8, CII-SDHB, CIII-UQCRC2, CIV-MTCO1, and CV-ATP5A) in the mitochondria (10 μg of protein per lane) isolated from the hearts of experimental rats. The analysis was carried out using the Total OXPHOS Rodent WB Antibody Cocktail (Abcam, #ab110413, Cambridge, UK) and polyclonal anti-VDAC1 antibodies (Abcam, #ab15895, Cambridge, UK). (**C**). Densitometric analysis of the content of the OXPHOS subunits in the heart mitochondria of rats from four experimental groups. Data are expressed as mean values ± standard deviations of three independent experiments. * *p* < 0.05 compared to the control group (CTR). Control values are taken as 100%.

We next used Western blot analysis and a real-time qPCR to examine possible changes in the protein levels of OXYPHOS subunits and the ratio between mitochondrial DNA and nuclear DNA copy numbers in the heart mitochondria of experimental animals. Figure 7A illustrates the relative mitochondrial DNA copy number, which is an indirect biomarker of mitochondrial dysfunction and may be associated with incident heart failure [44,45], in the heart tissue of experimental rats. There was no difference in the indicator between the four animal groups.

In the Western blot analysis, the relative protein expression of typical subunits of complexes I, II, III, and IV of the respiratory chain (CI-NDUFB8, CII-SDHB, CIII-UQCRC2, and CIV-MTCO1, correspondingly) was similar among the experimental groups (Figure 7B,C and Figure S1). The protein expression of ATP5A, one of the subunits of ATP synthase (complex V) in the mitochondria, was 30% lower in the ISO group compared with the control, a difference that was significant (*p* < 0.05). In the ISO + U group, the protein level of the complex V subunit did not significantly differ from the control value.

### 2.6. Effect of Uridine Pretreatment on Oxidative Stress Markers and Ion Transport Systems of Isolated Heart Mitochondria in Experimental Animal Groups

**Figure 8:**
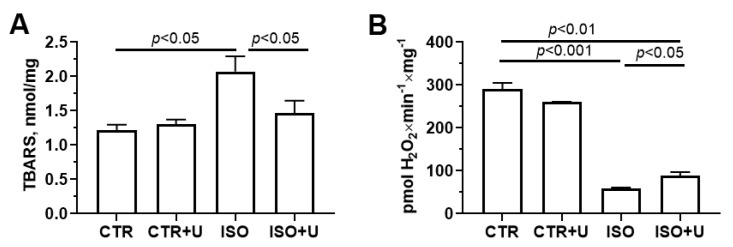
Effect of uridine pretreatment on oxidative stress-related parameters of isolated heart mitochondria of rats with ISO-induced cardiomyopathy. (**A**). TBARS levels in rat heart mitochondria from four experimental groups. (**B**). Rates of hydrogen peroxide production in rat heart mitochondria measured using the fluorescent dye Amplex Red. Values are mean ± SEM from a group of five animals. Statistical significance was analyzed using one-way ANOVA followed by Tukey’s post hoc test.

To investigate whether oxidative damage to mitochondria was increased in the ISO group, we measured mitochondrial markers of oxidative stress. Figure 8 shows the effect of uridine supplementation on the rate of hydrogen peroxide production and the content of TBA-reactive substances (mainly, the end product of oxygen radical-induced lipid peroxidation malondialdehyde) in the heart mitochondria of ISO-treated animals. One can see that, compared with the control, the rate of formation of hydrogen peroxide and the level of malondialdehyde in the heart mitochondria of rats in the ISO group increased by 27% and 21%, respectively. These indicators were lower in experimental animals co-treated with uridine (the ISO + U group) compared to the ISO group.

**Figure 9:**
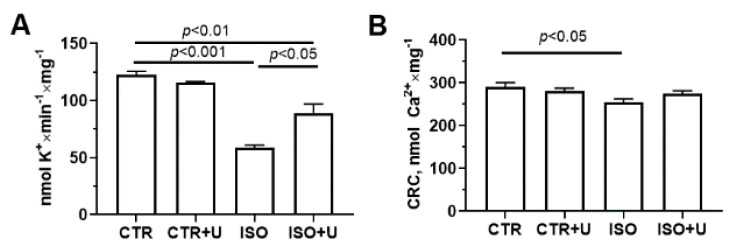
Effect of uridine pretreatment on potassium transport and calcium retention capacity (CRC) in isolated heart mitochondria of rats with ISO-induced cardiomyopathy. (**A**) Rate of release of potassium ions from cardiac mitochondria of experimental rats. (**B**) Mitochondrial ability to retain calcium ions in the matrix (the CRC index), indicating the resistance of the organelles to permeability transition pore formation. Mitochondrial ion transport was assessed using ion-selective microelectrodes. Values are mean ± SEM from a group of five animals. Statistical significance was analyzed using one-way ANOVA followed by Tukey’s post hoc test.

To comprehensively characterize the functional activity of mitochondria isolated from the hearts of experimental animals, in the next part of the work we studied changes in key ion transport systems. As one can see from Figure 9, the rate of potassium transport in mitochondria in the ISO group decreased two-fold compared to the control. The administration of uridine to ISO-treated animals (ISO + U group) resulted in an increase in the rate of mitochondrial potassium ion transport by 33%. In addition, the calcium retention capacity (CRC) index, which indicates the ability of mitochondria to retain calcium ions in the matrix and their invulnerability to the permeability transition pore (PTP) opening, was lower in the ISO group compared to the control. There was no difference in the CRC index between the ISO and ISO + U groups. It should be noted that in the CTR + U group, no difference in the CRC and oxidative stress-related parameters was observed compared to the control.

## 3. Discussion

This study was designed to investigate the effect of the metabolite uridine, which can promote tissue repair and regeneration in vivo, on changes in mitochondrial ultrastructure and functions after acute myocardial injury induced by high-dose ISO exposure in rats. In this in vivo model, two injections of ISO at a dose of 150 mg/kg body mass for two consecutive days caused profound myocardial damage, which was confirmed by biochemical and electrophysiological profiles. We first demonstrated the occurrence of urgent ultrastructural and functional alterations in mitochondria in rat cardiomyocytes as early as 48 h post-ISO, while preliminary treatment with uridine significantly reversed ISO-induced pathological changes in mitochondria. Uridine (30 mg/kg body mass, i.p.) was able to preserve mitochondrial energy, ionic, and redox metabolism in the cardiomyocytes of ISO-injected rats, suggesting that, in this aspect, mitochondrial dysfunction plays an important role in the onset of myocardial damage induced by excessive levels of catecholamines. Our findings may contribute to the development of preventive strategies for stress-induced myocardial injury.

Uridine, a pyrimidine nucleoside, is more abundant in plasma than other purine and pyrimidine nucleosides and its concentration in the blood is strictly regulated [30,46]. It is essential for the glycosylation process of proteins and lipids and the synthesis of endogenous pyrimidine and membrane phospholipids, being an obligatory precursor for CDP-choline synthesis [47]. As a precursor of UDP-glucose, uridine can activate the synthesis of glycogen for intracellular storage [27,28]. Uridine is involved in cellular signaling through P2Y receptors and can modulate systemic metabolism [47]. Recent data revealed that a higher level of plasma uridine reduces the tendency for atrial fibrillation and can be associated with a decreased atherosclerosis risk [48,49]. Our previous studies indicated that under ischemia and reperfusion conditions, uridine supplementation prevents myocardial redox dyshomeostasis and heart rhythm disorders [29,30]. A growing body of evidence suggests that uridine can induce tissue recovery and decrease pro-inflammatory cytokine levels in various models of cardiac injury and cell pathologies [32,33]. It is noteworthy that mitochondrial ROS are activated under a number of pathological and stress conditions, including β-adrenergic receptor-mediated heart damage, which in turn promotes increased expression of fibrotic genes in cardiac tissue [50]. Uridine treatment can significantly decrease the levels of oxidative stress and inflammation in in vitro and in vivo models [33,51]. The positive effects of uridine against myocardial oxidative stress and inflammation may be associated with the activation of the mitoK_ATP_ channel and normalization of the activity of antioxidant defense systems [29,30,33].

Here, we found that the exposure of rats to ISO causes a remarkable increase in the heart weight index, serum cardiac markers and lipid peroxidation products, along with electrophysiological disorders and ATP depletion in cardiac tissue. Altogether, these findings confirm that exposure to high-dose ISO causes pronounced myocardial damage in the acute (48 h) period and may serve as a suitable model for cardiomyopathy associated with energy and oxidative imbalances in rat cardiomyocytes. A number of studies have revealed a link between oxidative stress and catecholamine-induced cardiotoxicity [52,53,54]. Moreover, it is well established that mitochondrial dysfunction can contribute to the detrimental overproduction of ROS and impairment of ATP synthesis required for fueling myofilament contraction and ion transport [55]. Using the ISO overstimulation model, we show that uridine is capable of restoring mitochondrial ultrastructure and function in the context of acute cardiomyocyte injury. In our experimental setting, the dose of uridine was evaluated based on our previous studies on other models of pathologies associated with oxidative stress and the data available in the literature [29,30,33,34,35,56]. This dosage was well tolerated by control rats, without side effects during the follow-up period.

First, we compared electrophysiological and biochemical changes in rat blood serum and heart tissue to verify acute damage to the myocardium in the experimental groups. Electrophysiological examination revealed ISO-induced bradycardia and the prolongation of the most common intervals on the ECG, the QT and QRS complexes, which indicates an overall impairment of myocardial contractility and relaxation and is characteristic for this type of severe lesion [42,43,57]. Moreover, inversion of the T wave and a decrease in the amplitude of the R wave in the ISO group may indicate myocardial ischemia in the areas covered by the corresponding leads. That is, the process of repolarization (relaxation) of the ventricles is disrupted. The QT interval is a global reflection of all the electrical activity in the heart ventricles from the beginning of depolarization to the end of repolarization, while the QRS complex represents ventricular depolarization and activation. Many studies have revealed a cause–effect relationship between the prolongation of the QT interval and cardiac arrhythmias. Abdel-Hady et al. demonstrated that rats subjected to ISO-induced myocardial injury (85 mg/kg ISO/day for two successive days at a 24h interval) developed a high incidence of mortality and arrhythmia, together with bradycardia, and systolic and diastolic dysfunctions. [57]. Our data showed that pretreatment with uridine at a dose of 30 mg/kg 1 h before each injection of ISO results in a partial normalization of the QT interval, suggesting the alleviation of intraventricular conduction disorders.

A biochemical analysis revealed a sharp increase in the concentration of the cardiac-specific marker troponin I in the ISO group, along with the additional classical markers of cardiac injury, aspartate aminotransferase and alanine aminotransferase, and the content of the byproducts of lipid peroxidation in the serum. When uridine was administered to animals with ISO-induced cardiomyopathy, the levels of these biomarkers in the blood circulation significantly decreased, suggesting an alleviation of myocardial damage and a partial preservation of the structure of cardiomyocytes. In particular, pretreatment with uridine substantially reversed the ISO-induced 2.5-fold increases in the de Ritis ratio (AST/ALT), which is considered as a cardiometabolic marker and commonly used to help differentiate various causes of myocardial damage [58,59]. Furthermore, uridine administration maintained the level of ATP in the tissue of the left ventricle in ISO-injected rats, which may indicate a decrease in energy deficit and improvement in cardiac function in the failing hearts of animals. It is well known that disturbances in cardiomyocyte energy metabolism contribute to the severity of heart failure [17]. Some studies have reported that uridine can stimulate the conversion of its ribose-1-phosphate moiety into energetic intermediates of glycolysis, thereby replenishing glycogen reserves and preserving the ATP pool in cells [27,28]. Moreover, our results and literature data showed that, under hypoxic conditions, uridine preserves the morphofunctional organization of mitochondria [34], the main suppliers of ATP in cardiomyocytes via oxidative phosphorylation.

A morphometric analysis of electron microscopy images of rat cardiomyocytes revealed that the area of mitochondria in the ISO group was higher, indicating mitochondrial swelling, while pretreatment with uridine normalized the size of cardiac mitochondria. Moreover, uridine rescues mitochondrial integrity, the architecture of the cristae membranes, and the shape of the organelles in rat cardiomyocytes after acute exposure to ISO. It is noteworthy that the ISO + U group showed an increase in mitochondrial number. Previously, we found that uridine could prevent ultrastructural abnormalities and stimulate mitochondrial biogenesis by enhancing *Ppargc1a* expression in a mouse model of diabetic cardiomyopathy [35].

To comprehensively characterize the functional activity of mitochondria in the hearts of experimental animals, we then analyzed changes in oxidative phosphorylation, mitochondrial oxidative stress-related parameters, and key ion transport systems. In the ISO group, the ADP-stimulated respiration rate of mitochondria, the RCR ratio, and the ADP/O coefficient decreased in the presence of substrates of the respiratory chain complexes I and II, which may indicate a complex disorder of the oxidative phosphorylation system. Our results are consistent with the literature data obtained using similar ISO doses [15]. Uridine pretreatment significantly increased all the indexes of the efficiency of oxidative phosphorylation in rat heart mitochondria after acute ISO exposure. The underlying mechanism of this effect of uridine may be associated with an increase in the level of protein subunits of the respiratory chain complex V, ATP synthase, in the heart mitochondria of experimental rats in the ISO + U group. Recent evidence also suggests that uridine can regulate the functioning of the mitochondrial respiratory complexes via the activity of dihydroorotate dehydrogenase, which is coupled to the respiratory chain through coenzyme Q [36,37].

To investigate whether oxidative stress is increased in cardiac mitochondria in the ISO group, we measured markers of mitochondrial oxidative stress such as the rate of hydrogen peroxide production and the content of TBA-reactive substances (mainly, the end product of lipid oxidative damage, malondialdehyde). It was found that ISO increases the levels of ROS production and lipid peroxidation in the heart mitochondria of rats, while uridine pretreatment normalizes these indicators, suggesting the antioxidant potential of the compound. It is known that mitochondria are one of the main producers of ROS within most cells. Some studies have reported that ROS levels were elevated in samples of endomyocardial biopsy from patients with Takotsubo syndrome [3,5,7]. Notably, excess catecholamines are highly unstable and can undergo autoxidation to form reactive intermediates, which subsequently leads to the intracellular accumulation of lipid peroxides [4].

Another important aspect of mitochondrial functioning in cardiomyocytes is their involvement in the maintenance of intracellular calcium homeostasis [55]. In our experimental settings, ISO significantly reduced the mitochondrial CRC index, indicating a decrease in the ability of mitochondria to retain calcium ions in the matrix and their increased invulnerability to the PTP opening. The formation of the calcium-dependent PTP in heart mitochondria is a key event in the initiation of cell death in the most common causes of heart failure, including myocardial infarction, coronary heart disease, congenital heart defects, and others [19]. Yogeeta et al. reported that increased calcium concentrations in mitochondria can disturb the proton gradient across the inner mitochondrial membrane, thereby depleting ATP production in ISO-treated rats [60]. Our results indicate that uridine does not affect this indicator. 

We have previously shown that the uridine derivative 5′-uridine diphosphate is an activator of the mitoK_ATP_ channel [61] and that upon administration of uridine, the concentration of this activator in the blood and tissues increases [30,33]. Since uridine, unlike 5′-uridine diphosphate, penetrates into the cell, which additionally leads to the activation of the channel, we studied the effect of the nucleoside on potassium transport in the heart mitochondria of rats of the studied groups. In our experimental conditions, ISO significantly reduced the rate of mitochondrial transport of potassium ions, and prophylactic administration of uridine resulted in an increase in this parameter. According to our previous data and literature, this may be related to the activation of potassium recycling and a mild uncoupling of mitochondrial respiration [62,63]. The latter could be protective against oxidative injury by preventing the overproduction of ROS in the respiratory chain and ameliorating mitochondrial dysfunction and subsequent structural and functional damage to cardiomyocytes.

## 4. Materials and Methods

### 4.1. Chemicals

The chemicals used were purchased from Sigma-Aldrich (St. Louis, MO, USA), unless otherwise specified. Isoprenaline hydrochloride (Sigma-Aldrich, St. Louis, MO, USA; CAS 51-30-9, purity > 98.5%) was dissolved using normal saline to a concentration of 100 mg/mL to achieve a dose of 1.5 mL/kg. Uridine (PanReac AppliChem (ITW Reagents), Darmstadt, Germany; CAS 58-96-8, purity min. 99%) was dissolved in saline to a concentration of 60 mg/mL for intraperitoneal injections at a dose of 0.5 mL/kg.

### 4.2. Experimental Rats and Housing

The experimental procedures with animals used in this study are in line with the principles and practices of the 1986 European Guide for the Care and Use of Laboratory Animals in accordance with the Ethical Guidelines from the European Community Council Directive (86/609/EEC). All animal procedures were approved by the ITEB RAS Institutional Bioethics Committee at Pushchino, Moscow region, Russia (Protocol #01/2023 from 8 February 2023). Briefly, Wistar rats (12–13 weeks old, 200–230 g) were obtained and housed, five individuals in each cage, at the Animal Care Center of ITEB RAS under controlled conditions at 22 ± 2 °C room temperature, 55 ± 5% humidity with a 12 h light/12 h dark cycle. The animals were given free access to drinking water and rat chow for the standard diet (complete granular feed, recipe PK-120 for breeding laboratory animals, Laboratorkorm LLC, Moscow, Russia). The rats (*n* = 44) were randomly assigned to four groups, which consisted of 10–13 rats each. On the first and second days of the experiment, rats in the Isoprenaline (ISO; *n* = 13) and Isoprenaline + Uridine (ISO + U; *n* = 11) groups received 150 mg/kg/day of isoprenaline hydrochloride suspended in sterile saline subcutaneously (s.c.), and those in the Control (CTR; *n* = 10) and Uridine (CTR + U; *n* = 10) groups received an equivalent amount of saline solution. Animals in the CTR + U and ISO + U groups were pretreated intraperitoneally (i.p.) with 30 mg/kg of uridine solution. Uridine pretreatment in the ISO + U group was carried out by intraperitoneal injection one hour before each isoprenaline application. After three days, five rats from each group were fasted overnight, anesthetized with an intraperitoneal injection of a mixture of Zoletil (Virbac, France; 50 mg/kg) and Rometar (Bioveta, Czech Republic; 10 mg/kg), and monitored for heart failure symptoms using electrocardiogram (ECG) analysis. Animals were then sacrificed by decapitation, and the final body and heart weights were recorded. The heart tissue samples were snap-frozen and stored at −80 °C for ATP quantification or, alternatively, fixed for transmission electron microscopy (TEM) examination. The hearts of the remaining animals were used to isolate mitochondria and measure their functional activity. Blood samples from the rats were collected. Tubes with whole blood were preincubated at 37 °C for 1 h and then centrifuged using an Eppendorf Minispin centrifuge (Eppendorf, Hamburg, Germany) at 2000× *g* for 10 min to collect serum. The serum was stored at −20 °C until further assays.

### 4.3. Serum Cardiac Markers and Lipid Peroxidation Index

To evaluate the severity of myocardial damage, the concentration of the cardiac biomarker troponin I in the serum of experimental rats was determined by enzyme-linked immunosorbent assay using a quantitative Rat Cardiac Troponin I Kit (Cat. N ab246529, Abcam, Eugene, OR, USA). Additional markers of cardiac damage (AST and ALT) were measured spectrophotometrically using appropriate commercial kits (Cat. N B-8080, B-8078, ZAO Vector-Best, Novosibirsk, Russia). For the analysis, the blood serum was preincubated using a programmable solid-state thermostat (“Gnom”, Moscow, Russia) at 37 °C for 30 min. Further assay procedures were carried out as suggested by the manufacturers of the kits. The index of lipid peroxidation, thiobarbituric acid reactive substances (TBARS), was estimated using 1% TBA and 0.05 M sodium hydroxide (NaOH) with the help of a commercial kit (AGAT-Med Ltd., Moscow, Russia) and measured at two wavelengths—535 and 570 nm—in accordance with the manufacturer’s instructions.

### 4.4. Electrocardiographic Analysis

Electrocardiogram (ECG) measurements and analysis were performed in rats anesthetized with an intraperitoneal injection of a mixture of Zoletil (Virbac, France; 50 mg/kg) and Rometar (Bioveta, Czech Republic; 10 mg/kg). The animals were monitored in the supine position with the standard limb lead II and a Poly-Spectrum-8/B Veterinary Digital ECG System (NeuroSoft, Ivanovo, Russia) for 10 min. ECG recordings were analyzed using Spectrum 8/B hardware–software with veterinary-specific algorithms (NeuroSoft, Ivanovo, Russia).

### 4.5. Heart Tissue Homogenization and ATP Assay

The ATP content in heart tissue homogenates was measured using an ATP Fluorometric Assay Kit (Cat. N MAK190-1KT, Sigma-Aldrich, St. Louis, MO, USA) on a Spark 10M microplate reader (Tecan, Männedorf, Switzerland). After excision, the animal’s heart (the apex of the left ventricle) was snap-frozen and stored at −80 °C. Later on, the lysate was prepared by grinding the tissue in liquid nitrogen, followed by mixing the frozen homogenized tissue (10 mg) with 100 µL of ATP assay buffer and deproteinization using a Vivaspin 500 centrifugal concentrator (Cat. N VS0102, Sartorius AG, Goettingen, Germany). The lysate samples (5 µL) were then processed according to the manufacturer’s recommendations, using a set of standard ATP samples in a range of concentrations to create a calibration curve. The level of ATP was estimated by fluorescence detection (λex = 535 nm/λem = 587 nm) and expressed in nmol/mg of wet heart tissue weight.

### 4.6. Transmission Electron Microscopy Analysis

To identify structural damage to rat cardiomyocytes, identical sections of the left ventricle of the heart (subendocardial areas of the myocardium) were analyzed using transmission electron microscopy (TEM). Samples of the left ventricle fragments (three samples per group) were fixed with a 2.5% glutaraldehyde solution in 0.1 M of PBS buffer (pH 7.4) for 3 h at room temperature. Then, the samples were transferred to an osmic acid solution (1% *w*/*v*) and treated by increasing the concentration of alcohol in the alcohol/water mixture for dehydration. Subsequently, the samples were encapsulated in the polymerization resin Epon-812. A series of every five consecutive 60 nm thick sections was obtained using a Leica EM UC6 microtome (Leica Microsystems, Wetzlar, Germany). Each of the adjacent sections was stained with uranyl acetate and lead citrate according to the standard method and visualized using a JEM-100B electron microscope (JEOL, Tokyo, Japan). An Epson Perfection V700 Photo scanner was used to digitize the negatives. Morphometric analysis was performed using Image Tool 3.0 software to measure the average mitochondrial area and to count the average number of mitochondria in rat cardiomyocytes, normalized to the area (mm^2^) of the analyzed images.

### 4.7. Isolation of Rat Heart Mitochondria

Rat heart mitochondria were isolated from the tissue according to the standard method described in [35]. Briefly, the heart was excised and homogenized in a medium containing 75 mM sucrose (A2211, ITW Reagents, Gamburg, Germany), 225 mM mannitol (M4125, Sigma St. Louis, MO, USA), 0.5 mM EDTA (SKU04800682, MP Biomedicals LLC, Solon, OH, USA), 0.5 mM EGTA (E3889, Sigma St. Louis, MO, USA), 0.1% BSA fatty acid-free (A6003, Sigma St. Louis, MO, USA), and 10 mM Tris-HCl (pH 7.4). The isolation procedures were carried out at 4 °C. The heart tissue homogenate was precipitated at 1000× *g* for 10 min. The final supernatant was then centrifuged at 8500× *g* for 10 min. The pellet containing heart mitochondria was washed with an isolation medium without EDTA and BSA under the same centrifugation conditions and then resuspended. The Bradford method was applied to define the mitochondrial protein concentration, which was 35–40 mg/mL.

### 4.8. Evaluation of Mitochondrial Function

Mitochondrial oxygen consumption and oxidative phosphorylation were assessed by high-resolution respirometry using the Oroboros Oxygraph-2k with DatLab 4 software (Oroboros Instruments, Innsbruck, Austria) [35]. The incubation medium contained 130 mM KCl, 5 mM NaH_2_PO_4_, 10 µM EGTA, and 10 mM HEPES-KOH (pH 7.4). The respiration of mitochondria (0.5 mg/mL) was fueled by 2.5 mM potassium glutamate and 2.5 mM malate or 5.0 mM potassium succinate (in the presence of 1 µM rotenone). The energetic states assessed in rat heart mitochondria were as follows: state 2, basal substrate respiration (proton leak); state 3, respiration stimulated by 200 µM ADP; state 4, the mitochondrial state after all ADP is depleted; state 3U_DNP_, mitochondrial respiration in the presence of the uncoupling agent 2,4-dinitrophenol (DNP, 50 µM). The oxygen consumption rate was expressed as nmol O_2_/min·mg of protein. 

Mitochondrial ion transport was assessed in a temperature-controlled electrode chamber using calcium- and potassium-selective microelectrodes (NicoAnalyte LLC, Moscow, Russia) connected to a computerized multichannel electrometric system Record 4 (Pushchino, Russia). The mitochondrial potassium transport was calculated from the DNP-induced change in external K^+^ per one minute in the incubation medium containing 180 mM of sucrose, 70 mM of mannitol, 5 mM of NaH_2_PO_4_, 1 μg/mL of oligomycin, and 10 mM of Tris/HCl (pH 7.4), and 0.25 mg of mitochondrial protein/mL. The rate of potassium transport (nmol K^+^/min·mg of protein) across the mitochondrial membrane was assessed by adding 50 μM of DNP in each experiment. To determine the mitochondrial ability to retain calcium ions in the matrix (the calcium retention capacity index), indicating the resistance of the organelles to the mPTP formation, 2.5 μM CaCl_2_ pulses were applied to the medium containing 210 mM mannitol, 70 mM sucrose, 1 mM KH_2_PO_4_, 2.5 mM potassium glutamate, 2.5 mM potassium malate, 10 μM EGTA, and 10 mM HEPES-KOH (pH 7.4). The CRC index was calculated as the amount of calcium ions taken up by the organelles (0.25 mg of mitochondrial protein/mL) in pulse additions before a massive calcium release. 

To assess the level of lipid peroxidation in rat heart mitochondria, the content of TBARS was estimated using a commercial kit (AGAT-Med Ltd., Moscow, Russia) and measured at two wavelengths—535 and 570 nm—in accordance with the manufacturer’s instructions [35]. The generation of H_2_O_2_ by mitochondria was registered with the fluorescent probe Amplex Red (λex = 560 nm; λem = 590 nm) using the plate reader Tecan Spark 10M at 37 °C, as previously described [60]. Rat heart mitochondria (0.12 mg/mL) were incubated in the medium containing 70 mM sucrose, 210 mM mannitol, 2.5 mM malate, 2.5 mM glutamate, 1 mM KH_2_PO_4_, 1 a.u./mL horseradish peroxidase, 10 µM Amplex Red, and 10 mM HEPES-KOH (pH 7.4). The amount of the generated H_2_O_2_ was calculated using a standard curve prepared with fresh H_2_O_2_ solutions of the known concentrations.

### 4.9. Mitochondrial DNA Copy Number

Quantitative real-time PCR was used to estimate the mtDNA content in the samples using a QuantStudio 1 amplifier (Thermo Fisher Scientific, Waltham, MA, USA) and a qPCRmix-HS SYBR reaction kit containing SYBR Green PCR Master Mix (Eurogen, Moscow, Russia), as previously reported [35]. Samples of the left ventricle myocardium (subendocardial areas, six samples in each experimental group) were taken from decapitated rats for total (nuclear and mitochondrial) DNA isolation using the DNA-Extran-2 kit (Sintol LLC, Moscow, Russia). The data were calculated as the ratio of the mitochondrial DNA copy number to the nuclear DNA copy number; the results were then normalized to the control values. For the analysis, the *ND4* gene encoded by the mitochondrial genome and the *GAPDH* gene of the nuclear genome were selected. Oligonucleotide sequences for forward and reverse primers were as follows: *Gapdh* (glyceraldehyde-3-phosphate dehydrogenase) forward, TGGCCTCCAAGGAGTAAGAAAC; *Gapdh* reverse, GGCTCTCTCCTTGCTCTCAGTATC; *mt-tRNA* forward, AATGGTTCGTTTGTTCAACGATT; *mt-tRNA* reverse, AGAAACCGACCTGGATTGCTC.

### 4.10. Electrophoresis and Immunoblotting

The samples of rat heart mitochondria (25–30 mg/mL) dissolved in Laemmli buffer (Bio-Rad, Hercules, CA, USA) were added to each line at 10 μg and separated by electrophoresis (12.5% SDS-PAGE). The proteins were transferred from the gel onto a 0.45-µm nitrocellulose membrane (Amersham, Germany). The membranes were blocked in 2% non-fat milk blocking buffer (PBS) for 24 h at 4 °C. After blocking, the membranes were stained with a total OXPHOS Rodent WB Antibody Cocktail (ab110413, Abcam, Cambridge, UK). Polyclonal anti-VDAC1 antibodies (ab15895, Abcam, Cambridge, UK) were used to normalize the protein. A commercial rat heart tissue lysate–mitochondrial extract (SDS/DTT) (ab110341, Abcam, Cambridge, UK) was used as a positive control (Supplementary Materials, Figure S1 (the abbreviation PC)). Immunoreactivity was determined using secondary antibodies conjugated with horseradish peroxidase (Cell Signaling technology Inc., Danvers, MA, USA). Peroxidase activity was detected with ECL chemiluminescence reagents (Bio-Rad, Hercules, CA, USA) using a LI-COR system (LI-COR, Lincoln, NE, USA) and were normalized by the ratio of proteins to VDAC1. Protein bands were quantified by densitometry (LI-COR Image Studio 5.2 software).

### 4.11. Statistical Analysis

Analyses were executed by using Excel (Microsoft Office Excel 2021; Microsoft, Richmond, CA, USA) and GraphPad Prism9 (GraphPad Software, San Diego, CA, USA). The Shapiro–Wilk test was used to check whether the variable data are normally distributed. Variable data were expressed as means ± standard error of the mean (SEM) with the number of rats unless otherwise indicated; categorical indexes were normalized to control values. For the data with a normal distribution, statistical significance was analyzed using one-way ANOVA followed by Tukey’s post hoc test. Statistical significance was considered to be the value of *p* < 0.05.

## 5. Conclusions

The present study provides the first insight into the use of uridine as a protective agent in acute stress-induced cardiomyopathy. Pretreatment with uridine was able to reverse ISO-induced changes in the serum biomarker troponin I and the De Ritis ratio, electrocardiographic disturbances, and structural alterations in cardiomyocytes and mitochondria. Uridine restored the efficacy of oxidative phosphorylation and the expression level of ATP synthase in mitochondria, thereby partially restoring the level of ATP in the myocardium. These beneficial effects of uridine are likely due to the attenuation of the signs of mitochondrial dysfunction observed in the pathology, which can contribute to the detrimental production of ROS and impair ATP synthesis, required not only for fueling myofilament contraction, but for ion transport as well. Uridine preserves the activity of potassium transport systems, which may promote potassium recycling in mitochondria. In turn, this may prevent excessive ROS production in the mitochondrial respiratory chain by the induction of a mild uncoupling, thereby ultimately ameliorating mitochondrial dysfunction and oxidative damage to the myocardium. The data obtained on the protective effect of uridine against mitochondrial dysfunction and myocardial injury in ISO overstimulation can be used to develop effective integrated approaches to the treatment of stress-related heart attack and heart failure. However, further studies are needed to identify the underlying mechanisms of the effect of uridine, both in acute and persistent myocardial injury in stress-induced cardiomyopathy.

## Figures and Tables

**Table 1:** Indices of respiration and oxidative phosphorylation of rat heart mitochondria in four experimental groups.

Group	V Respiration, nmol O_2_·min^−1^·mg^−1^ pr				RCR	ADP/O	T_ph,_ s
	State 2	State 3	State 4	State 3U_DNP_			
CTR	4.6 ± 0.5	67.4 ± 3.7	13.9 ± 0.8	63.8 ± 3.4	4.90 ± 0.22	2.96 ± 0.12	43.6 ± 2.8
CTR + U	4.2 ± 1.3	57.9 ± 1.2	12.5 ± 1.3	61.5 ± 3.3	4.67 ± 0.20	2.74 ± 0.07	42.0 ± 4.3
ISO	4.0 ± 0.8	45.4 ± 1.8 *	13.5 ± 1.0	42.9 ± 1.8 *	3.38 ± 0.18 *	2.18 ± 0.08 *	69.6 ± 3.9 *
ISO + U	3.9 ± 0.9	54.8 ± 2.1 *^,#^	13.1 ± 0.7	47.9 ± 2.6 *	4.20 ± 0.19 *^,#^	2.53 ± 0.10	49.7 ± 4.6 *^,#^

Medium composition: 130 mM KCl, 5 mM NaH_2_PO_4_, 10 µM EGTA, and 10 mM HEPES-KOH, pH 7.4. Respiration of mitochondria was fueled by 2.5 mM glutamate and 2.5 mM malate. Mitochondrial respiration in State 3 was initiated by 200 µM ADP. Mitochondrial respiration in state 3U_DNP_ was initiated by 50 µM 2,4-dinitrophenol. The results are presented as means ± SEM (*n* = 8–10). * *p* < 0.05 compared to the control group (CTR); ^#^ *p* < 0.05 compared to the ISO group.

## Data Availability

The data presented in this study are available upon request from the corresponding author.

## Supplementary Materials

The following supporting information can be downloaded at: https://www.mdpi.com/article/10.3390/ijms242417300/s1.
