# Supplementary material for: Protective Effect of Uridine on Structural and Functional Rearrangements in Heart Mitochondria after a High-Dose Isoprenaline Exposure Modelling Stress-Induced Cardiomyopathy in Rats
Source: Int J Mol Sci. 2023 Dec 9;24(24):17300. doi: 10.3390/ijms242417300 (PMC10744270; doi:10.3390/ijms242417300)
Supplement: Supplementary file 1 [file ijms-24-17300-s001.zip › ijms-2760851-supplementary.pdf]

Supplementary materials

to the manuscript of "Protective Effect of Uridine on Structural and Functional Rearrangements in Heart Mitochondria after a High-Dose Isoprenaline Exposure Modelling Stress-Induced Cardiomyopathy in Rats"

Table S1. Indices of the succinate-fueled respiration and oxidative phosphorylation of rat heart mitochondria in four experimental groups

**Group**	**V respiration, nmol O_2 * min^{-1} * mg^{-1} protein**				**RCR**	**ADP/O**	**T_{ph}, s**
	State 2	State 3	State 4	State $3U_{DNP}$			
CTR	28.4±4.9	136.3±7.8	43.5±4.2	137.0±10.8	3.13±0.18	1.99±0.09	22.8±3.7
CTR+U	24.9±1.7	112.9±7.2	37.0±1.3	114.5±13.3	3.06±0.19	1.98±0.07	26.6±3.9
ISO	20.9±3.6	84.2±6.4*	28.4±2.3*	85.4±6.2*	2.91±0.11	1.87±0.08	39.4±2.9*
ISO+U	21.6±3.9	87.7±7.5*	27.2±1.7*	93.3±5.6*	3.12±0.13	1.92±0.09	33.3±3.1

Medium composition: 130 mM KCl, 5 mM NaH_2PO_4, 10 μM EGTA, and 10 mM HEPES-KOH, pH 7.4. Respiration of mitochondria was fueled by 5.0 mM potassium succinate (in the presence of 1 μM rotenone). Mitochondrial respiration in State 3 was initiated by 200 μM ADP. Mitochondrial respiration in state $3U_{DNP}$ was initiated by 50 μM 2,4-dinitrophenol. The results are presented as means ± SEM (n = 8-10). $^{*}p < 0.05$ compared to the control group (CTR); $^{\#}p < 0.05$ compared to the ISO group.

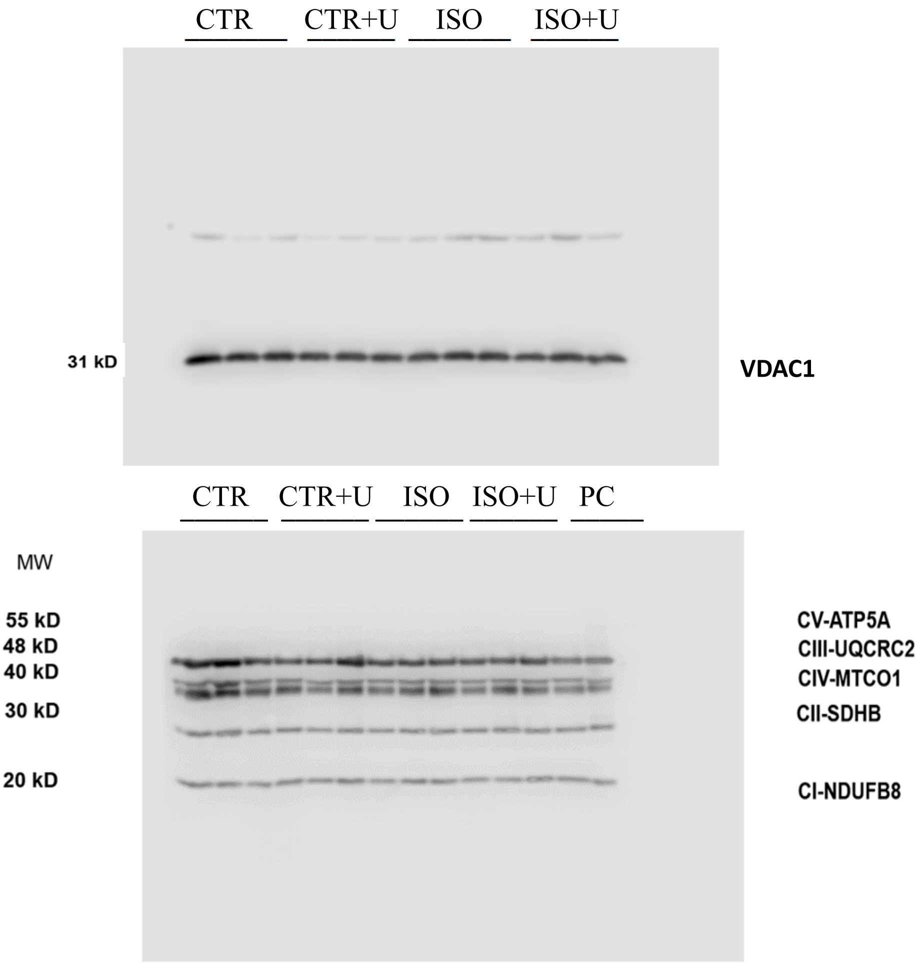

Figure S1. Representative Western blot of typical subunits of complexes I-V of the respiratory chain (CI-NDUFB8, CII-SDHB, CIII-UQCRC2, CIV-MTCO1 and CV-ATP5A) in the mitochondria isolated from the heart of experimental rats. Rat heart mitochondria were applied in an amount of 10 μg of protein per band. The analysis was carried out using the Total OXPHOS Rodent WB Antibody Cocktail (Abcam, #ab110413) and the polyclonal anti-VDAC1 antibodies (Abcam, #ab15895). Analysis of the content of the OXPHOS subunits in the heart mitochondria of rats from four experimental groups (three independent experiments). PC - positive control (Abcam, #ab110341) (10 μg of protein in each of the last two bands).
